# Prevalence and Clinical Characteristics of Pre-sarcopenia, Dynapenia, and Sarcopenia in Elderly Japanese Patients With Osteoporosis

**DOI:** 10.7759/cureus.110263

**Published:** 2026-06-04

**Authors:** Masayuki Miyagi, Akiyoshi Kuroda, Yuji Yokozeki, Kosuke Murata, Hisako Fujimaki, Yusuke Mimura, Yoshihide Tanaka, Shun Nokariya, Gen Inoue, Masashi Takaso

**Affiliations:** 1 Department of Orthopedic Surgery, Kitasato University School of Medicine, Sagamihara, JPN; 2 Orthopedic Surgery, Kitasato University, Sagamihara, JPN

**Keywords:** dynapenia, hand grip strength, low back pain (lbp), osteoporosis, skeletal muscle mass, sarcopenia

## Abstract

Background

Sarcopenia and dynapenia are age-related conditions characterized by reductions in skeletal muscle mass and/or muscle strength. Both conditions are associated with functional decline and impaired quality of life in elderly populations. This study investigated the prevalence and clinical characteristics of sarcopenia, pre-sarcopenia, and dynapenia in elderly patients with osteoporosis.

Methodology

This cross-sectional study included 332 elderly patients with osteoporosis, including 46 men and 286 women. Participants were classified into the following four groups according to skeletal muscle mass index and grip strength: normal, pre-sarcopenia, dynapenia, and sarcopenia groups. Bone mineral density, body composition, spinal sagittal alignment, vertebral fractures, and low back pain-related clinical outcomes were compared among groups.

Results

Pre-sarcopenia, dynapenia, and sarcopenia were identified in 42/332 (12.7%), 94/332 (28.3%), and 47/332 (14.2%) patients, respectively. Overall, 183/332 (55.1%) patients demonstrated reduced muscle mass and/or strength. Lower bone mineral density and body mass index were predominantly observed in groups with decreased muscle mass, whereas poorer low back pain-related outcomes were mainly associated with reduced muscle strength.

Conclusions

A substantial proportion of elderly patients with osteoporosis demonstrated impaired muscle mass and/or muscle strength. Muscle mass appeared to be associated with bone density and body composition, whereas muscle strength was more closely related to trunk muscle mass and low back pain-related disability.

## Introduction

Age-related decline in skeletal muscle function has become an important clinical issue in aging societies. Sarcopenia is generally defined as a progressive decrease in both skeletal muscle mass (SMM) and muscle strength [1-3], resulting in impaired physical performance and increased risk of disability [4]. In contrast, dynapenia refers primarily to reduced muscle strength despite relatively preserved muscle mass. Both conditions are increasingly recognized as major contributors to frailty and reduced independence among older adults [5]. Importantly, loss of muscle mass and loss of muscle strength do not always occur simultaneously. Some individuals exhibit reduced muscle mass despite preserved muscle strength (pre-sarcopenia), whereas others demonstrate reduced muscle strength despite relatively preserved muscle mass (dynapenia).

Osteoporosis is another common disorder in elderly populations and is characterized by decreased bone mass and increased fracture susceptibility. Previous studies have demonstrated close interactions between bone and muscle metabolism [6,7]. Furthermore, elderly patients with osteoporosis frequently experience spinal deformity, low back pain, and functional decline [8,9]. However, the respective influences of muscle mass and muscle strength on spinal alignment, bone mineral density (BMD), and clinical outcomes remain insufficiently understood. In particular, little is known about whether patients with pre-sarcopenia and dynapenia exhibit different clinical characteristics from those with established sarcopenia.

The goal of this study was to clarify the prevalence and clinical characteristics of sarcopenia, pre-sarcopenia, and dynapenia in elderly patients with osteoporosis and to determine whether muscle mass and muscle strength are associated with different clinical and radiographic characteristics.

## Materials and methods

The study was conducted in accordance with the Declaration of Helsinki and was approved by our Institutional Review Board (approval code: #B20-199).

This cross-sectional observational study included 332 elderly patients with osteoporosis who visited our institution. The study population consisted of 46 men and 286 women aged older than 65 years. Osteoporosis was diagnosed according to the Japanese Guidelines for the Prevention and Treatment of Osteoporosis 2025. Patients were diagnosed based on BMD and/or the presence of fragility fractures. Primary osteoporosis was diagnosed in 219 patients, while 113 patients had secondary osteoporosis related to glucocorticoid therapy, rheumatoid arthritis, diabetes mellitus, chronic kidney disease, or chronic obstructive pulmonary disease. Patients with severe standing difficulty, pacemakers, or metallic implants affecting bioelectrical impedance analysis were excluded.

BMD of the lumbar spine (LS), femoral neck (FN), and total hip (TH) was measured using dual-energy X-ray absorptiometry (Horizon DXA System; Hologic Inc., CA, USA). Nutritional status was evaluated using serum albumin, total cholesterol, lymphocyte counts, and the Controlling Nutritional Status (CONUT) score [10]. Bone turnover markers included bone-specific alkaline phosphatase (BAP) and tartrate-resistant acid phosphatase 5b (TRACP5b).

Whole-spine standing radiographs were obtained with both hands positioned on the clavicles. The number of vertebral fractures was assessed, and sagittal alignment parameters including pelvic tilt (PT), pelvic incidence minus lumbar lordosis (PI-LL), and sagittal vertical axis (SVA) were measured. PT was evaluated as the angle formed by a line connecting the midpoint of the sacral endplate and the bicoxofemoral axis relative to the vertical axis. PI was defined as the angle between a line perpendicular to the sacral endplate at its midpoint and a line connecting this point to the center of the femoral heads [11], whereas LL was measured between the superior endplates of L1 and S1. SVA was measured as the horizontal offset between the C7 plumb line and the posterior superior corner of the sacrum [12].

Body composition analysis was performed using bioelectrical impedance analysis MC-780 (Tanita Co., Tokyo, Japan). Evaluated parameters included body mass index (BMI), SMM, trunk muscle mass (TMM), skeletal muscle mass index (SMI), and trunk muscle mass index (TMI). SMI and TMI were calculated by normalizing SMM and TMM to height squared (kg/m²), respectively.

Low back pain-related outcomes were assessed using the Japanese Orthopaedic Association Back Pain Evaluation Questionnaire (JOABPEQ) and the Oswestry Disability Index (ODI). The JOABPEQ evaluates five domains, namely, pain-related disorders, lumbar spine dysfunction, gait disturbance, social functioning, and psychological status. Each domain is scored on a scale from 0 to 100, with higher values representing better functional status [13]. In contrast, higher ODI scores reflect greater disability and poorer clinical condition [14].

According to the Asian Working Group for Sarcopenia criteria 2019, decreased grip strength was defined as <28 kg in men and <18 kg in women. Decreased SMI was defined as <7.0 kg/m² in men and <5.7 kg/m² in women [3]. Participants were classified into four groups based on the presence or absence of reduced SMM and reduced grip strength. Individuals with neither reduced muscle mass nor reduced grip strength were classified as the normal group. Those with reduced muscle mass but preserved grip strength were classified as the pre-sarcopenia group, whereas those with preserved muscle mass but reduced grip strength were classified as the dynapenia group [15].

Categorical variables were compared among four groups using the chi-square test, and continuous variables were compared among four groups using one-way analysis of variance followed by Tukey’s post hoc test. Analyses of body composition and BMD were performed separately for male and female participants to account for sex-related differences in body constitution, and statistical significance was defined as a p-value <0.05. Associations between SMI or grip strength and BMD, radiographic parameters, or clinical outcomes were evaluated using Spearman correlation analysis in each sex independently. Correlation strength was interpreted as weak for coefficients between 0.2 and 0.4, moderate for values between 0.4 and 0.7, and strong for values greater than 0.7. Statistical analyses were performed using SPSS Statistics version 26 (IBM Corp., Armonk, NY, USA).

## Results

Among the 332 enrolled patients, 42 (12.7%) were classified as pre-sarcopenia, 94 (28.3%) as dynapenia, and 47 (14.2%) as sarcopenia. In men, the prevalence of pre-sarcopenia, dynapenia, and sarcopenia was 10/46 (21.7%), 11/46 (23.9%), and 8/46 (17.4%), respectively, whereas corresponding values in women were 32/286 (11.2%), 83/286 (29.0%), and 39/286 (13.6%), respectively. In addition, the proportion of primary and secondary osteoporosis differed among groups, suggesting that differences in underlying disease background may partially contribute to variations in muscle mass and muscle strength (Table 1).

**Table 1:** Baseline demographic and clinical characteristics of elderly patients with osteoporosis among four groups. The comparisons of categorical variables were performed using the chi-square test.

	Normal	Pre-sarcopenia	Dynapenia	Sarcopenia	Total
N	149	42	94	47	332
Men	20	10	11	8	49
Women	129	32	83	39	283
P-value					0.28
Primary	111	28	50	30	219
Secondary	38	14	44	17	113
P-value					0.008

**Figure 1:**
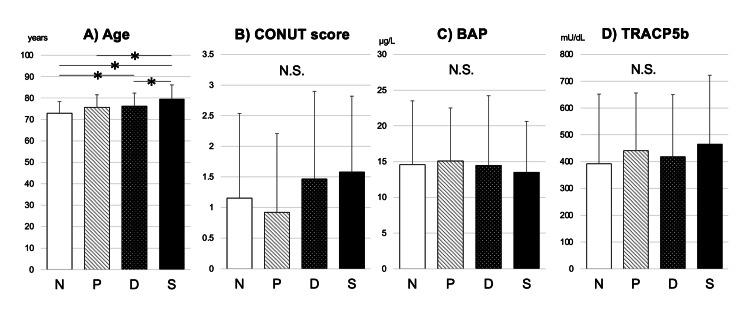
Multiple comparisons of (A) the mean age, (B) the CONUT score, and (C, D) bone turnover markers. Statistical analysis was performed using one-way analysis of variance, followed by Tukey’s post hoc test. *: P < 0.05. N = normal group; P = pre-sarcopenia group; D = dynapenia group; S = sarcopenia group; BAP = bone-specific alkaline phosphatase; TRACP5b = tartrate-resistant acid phosphatase 5b; CONUT = Controlling Nutritional Status; N.S. = not significant

Patients in the dynapenia and sarcopenia groups tended to be older than those in the normal group. Furthermore, the sarcopenia group demonstrated a higher mean age than the dynapenia and pre-sarcopenia groups (Figure 1A). In contrast, CONUT scores and bone turnover markers were generally comparable among the four groups (Figures 1B-1D).

**Figure 2:**
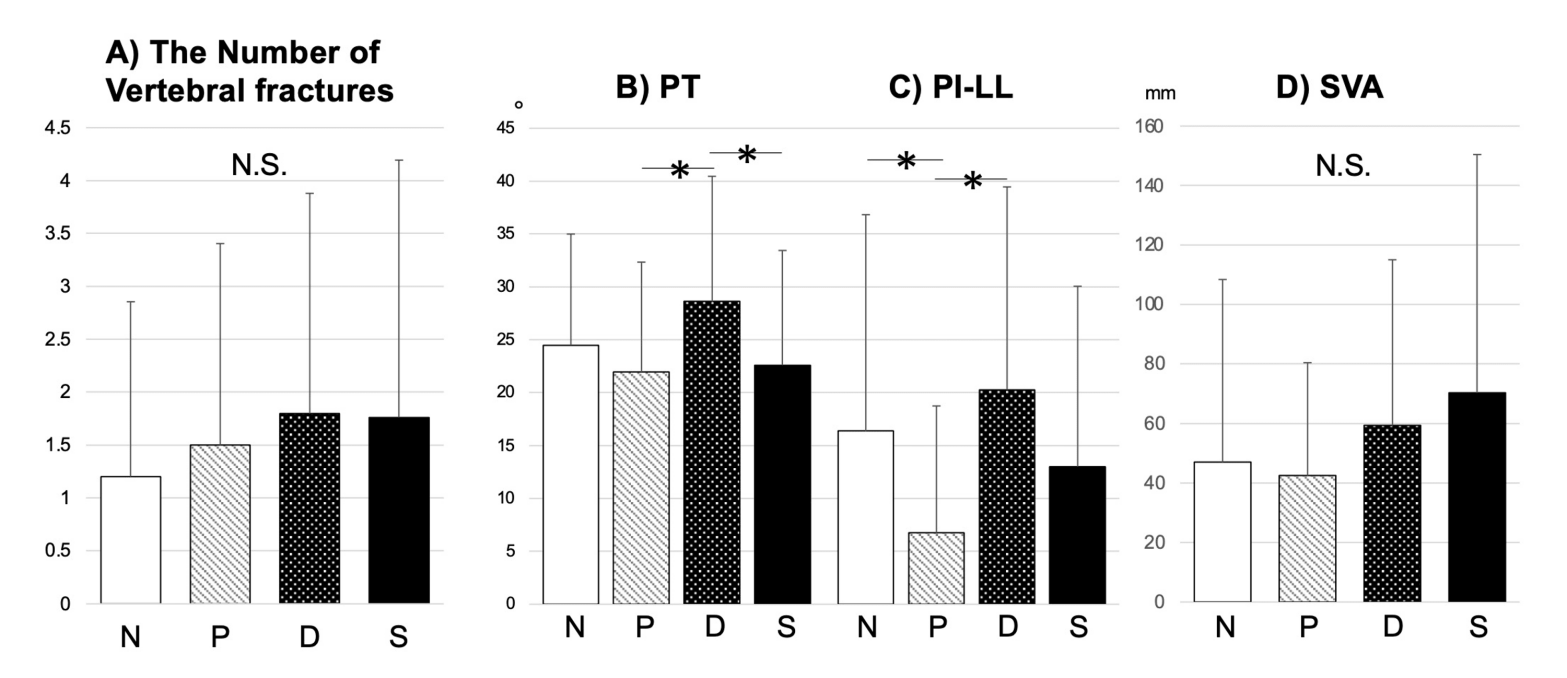
Multiple comparisons of (A) the number of vertebral fractures and (B, C, D) spinal sagittal alignment parameters. Statistical analysis was performed using repeated-measures analysis of variance followed by Tukey’s post hoc test. *: P-value <0.05. N = normal group; P = pre-sarcopenia group; D = dynapenia group; S = sarcopenia group; PT = pelvic tilt; PI-LL = pelvic incidence minus lumbar lordosis; SVA = sagittal vertical axis; N.S. = not significant

No marked differences in vertebral fracture number were identified among the groups (Figure 2A). Analysis of spinal sagittal alignment revealed that PT values were greater in the dynapenia group than in the sarcopenia and pre-sarcopenia groups (Figure 2B). PI-LL values were higher in the normal and dynapenia groups than in the pre-sarcopenia group (Figure 2C), whereas SVA values were similar across all groups (Figure 2D).

**Figure 3:**
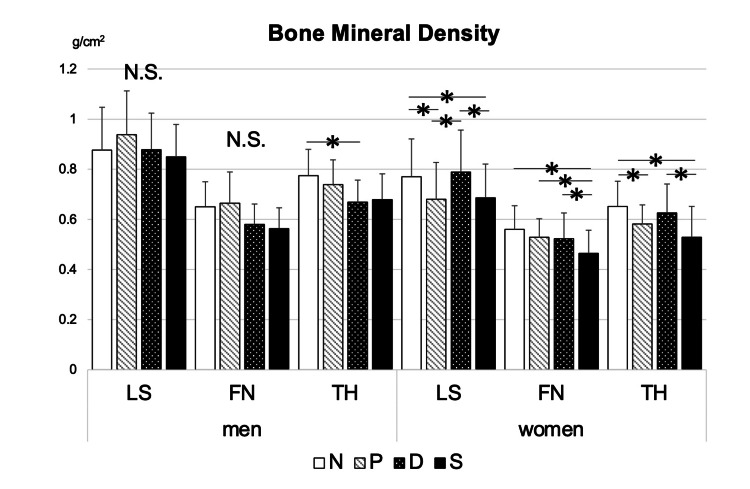
Multiple comparisons of the bone mineral density. Statistical analysis was performed using repeated-measures analysis of variance followed by Tukey’s post hoc test. *: P-value <0.05. N = normal group; P = pre-sarcopenia group; D = dynapenia group; S = sarcopenia group; LS = lumbar spine; FN = femoral neck; TH = total hip; N.S. = not significant

Sex-related differences were identified in BMD measurements. In men, LS and FN BMD values were largely similar among groups; however, TH BMD was lower in the dynapenia group than in the normal group. In women, LS BMD was reduced in the pre-sarcopenia and sarcopenia groups compared with the normal and dynapenia groups. FN BMD was the lowest in the sarcopenia group, and TH BMD was reduced in the pre-sarcopenia and sarcopenia groups relative to the normal group. In addition, women in the sarcopenia group demonstrated lower TH BMD than those in the dynapenia group (Figure 3).

**Figure 4:**
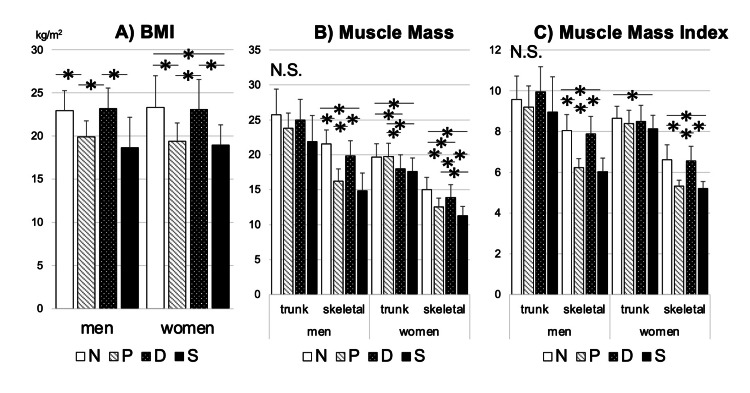
Multiple comparisons of (A) body composition including BMI, (B) muscle mass, and (C) muscle mass index. Statistical analysis was performed using repeated-measures analysis of variance followed by Tukey’s post hoc test. *: P-value <0.05. N = normal group; P = pre-sarcopenia group; D = dynapenia group; S = sarcopenia group; BMI = body mass index; N.S., not significant

Body composition analyses revealed similar tendencies for BMI, SMM, and SMI (Figures 4A-4C). Female patients in the sarcopenia and pre-sarcopenia groups had lower BMI values than those in the normal and dynapenia groups. Likewise, SMM and SMI were reduced in the sarcopenia and pre-sarcopenia groups in both sexes. In men, BMI was also lower in the sarcopenia and pre-sarcopenia groups than in the dynapenia group, and lower in the pre-sarcopenia group than in the normal group. Different findings were observed for TMM and TMI. No clear intergroup differences were identified for TMM or TMI in men. In women, however, TMM was lower in the dynapenia and sarcopenia groups than in the normal and pre-sarcopenia groups. TMI was also lower in the sarcopenia group than in the normal group among female participants.

**Figure 5:**
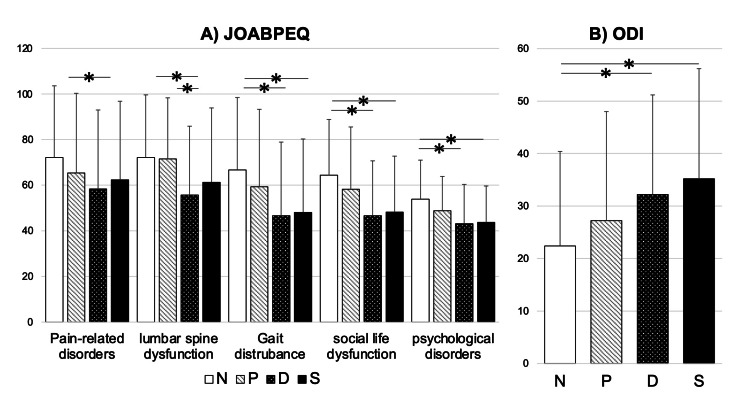
Multiple comparisons of the clinical outcomes including JOABPEQ (A) and ODI (B). Statistical analysis was performed using repeated-measures analysis of variance followed by Tukey’s post hoc test. *: P-value <0.05. N = normal group; P = pre-sarcopenia group; D = dynapenia group; S = sarcopenia group; JOABPEQ = Japanese Orthopedic Association Back Pain Evaluation questionnaire; ODI = Oswestry Disability Index

Clinical outcome measures demonstrated patterns similar to those observed for muscle strength. The dynapenia group had poorer pain-related disorder scores than the normal group. LS dysfunction scores were also worse in the dynapenia group than in the normal and pre-sarcopenia groups. In addition, gait disturbance, social life dysfunction, and psychological disorder scores were less favorable in the dynapenia and sarcopenia groups than in the normal group. By contrast, JOABPEQ scores in the pre-sarcopenia group were comparable to those in the normal group (Figure 5A). Similar tendencies were identified for ODI scores, with higher disability levels observed in the dynapenia and sarcopenia groups, whereas the pre-sarcopenia and normal groups showed comparable ODI results (Figure 5B).

The relationships among SMI, grip strength, BMD, radiographic parameters, and clinical outcomes are summarized in Table 2. In women, higher SMI values were associated with greater BMD at the LS, FN, and TH, although the strength of these associations was limited. Aside from LS dysfunction scores in men, SMI was not meaningfully related to spinal alignment parameters or patient-reported clinical outcomes. Grip strength exhibited broader associations than SMI. Positive relationships were identified between grip strength and FN or TH BMD in both sexes, whereas LS BMD in women did not correlate significantly with grip strength. In male patients, weaker but significant associations were also identified between grip strength and sagittal alignment measurements, including PT, PI-LL, and SVA. Patient-reported outcomes were more closely linked to muscle strength than muscle mass. Lower grip strength was associated with poorer JOABPEQ and ODI scores across most domains. The only exception was the pain-related disorder domain of the JOABPEQ in women, which did not demonstrate a statistically significant relationship with grip strength.

**Table 2:** Correlations between skeletal muscle mass index or grip strength and bone mineral density or radiographical findings or clinical outcomes Correlations were analyzed using Spearman’s correlation coefficients. Statistical significance was defined as a p-value <0.05 as well as r >0.200, which are indicated by an asterisk (*). SMI = skeletal muscle mass index; BMD = bone mineral density; LS = lumbar spine; FN = femoral neck; TH = total hip; PT = pelvic tilt; PI-LL = pelvic incidence minus lumbar lordosis; SVA = sagittal vertical axis; PrD = pain-related disorders; LSD = lumbar spine dysfunction; GD = gait disturbance; SLD = social life dysfunction; PlD = psychological disorders; JOABPEQ = Japanese Orthopedic Association Back Pain Evaluation questionnaire; ODI = Oswestry Disability Index

			BMD			Radiographical findings			JOABPEQ					ODI
			LS	FN	TH	PT	PI-LL	SVA	PrD	LSD	GD	SLD	PlD	
SMI	Men	r	0.076	0.171	0.268	0.093	0.076	0.014	-0.129	-0.350	0.009	0.026	-0.040	0.004
		p	0.601	0.246	0.065	0.569	0.642	0.934	0.387	0.016*	0.950	0.861	0.788	0.977
	Women	r	0.303	0.248	0.374	0.150	0.197	0.199	-0.007	-0.032	-0.089	-0.039	-0.010	-0.036
		p	0.000*	0.000*	0.000*	0.022	0.003	0.002	0.906	0.592	0.137	0.513	0.869	0.553
Grip strength	Men	r	0.237	0.456	0.533	-0.397	-0.298	-0.254	0.335	0.373	0.575	0.523	0.356	-0.412
		p	0.101*	0.001*	0.000*	0.011*	0.062*	0.114*	0.021*	0.010*	0.000*	0.000*	0.014*	0.004*
	Women	r	-0.026	0.301	0.261	-0.113	-0.093	-0.042	0.158	0.279	0.310	0.344	0.329	-0.300
		p	0.661	0.000*	0.000*	0.086	0.157	0.525	0.008	0.000*	0.000*	0.000*	0.000*	0.000*

## Discussion

In the present cohort, pre-sarcopenia, dynapenia, and sarcopenia were identified in 12.7%, 28.3%, and 14.2% of patients with osteoporosis, respectively. Notably, more than half of the study population (55.1%) demonstrated reduced muscle mass and/or muscle strength. In addition, patients classified into the pre-sarcopenia, dynapenia, and sarcopenia groups were older than those in the normal group. Furthermore, the proportion of secondary osteoporosis was significantly higher in these groups than in the normal group, suggesting that differences in underlying disease background may be associated with variations in muscle mass and muscle strength. Previous studies have reported similar findings in elderly Japanese populations. Yamada et al. described prevalences of 11.0% for pre-sarcopenia, 29.4% for dynapenia, and 23.3% for sarcopenia in an older Japanese cohort [15]. Hori et al. demonstrated age-related reductions in both SMM and TMM among patients with spinal disorders [16]. Likewise, Curtis et al. suggested that aging negatively influences muscle quantity, quality, and physical performance [17]. Goodpaster et al. further reported that preservation of muscle mass alone may not be sufficient to prevent age-associated decline in muscle strength [18]. Taken together, these findings suggest that impairment of muscle mass and muscle strength is highly prevalent in elderly patients with osteoporosis. Aging may therefore contribute not only to quantitative muscle loss but also to deterioration in muscle quality and function.

In the present study, no clear intergroup differences were identified with respect to vertebral fracture number or spinal sagittal alignment parameters. Previous investigations have suggested that reduced TMM and decreased grip strength are independently associated with spinal sagittal malalignment in patients with spinal disorders [19]. However, earlier studies also raised concerns regarding insufficient evaluation of vertebral fractures when assessing spinal alignment [19]. Several reports have emphasized the close relationship between vertebral fractures and sagittal balance in patients with osteoporosis. Langella et al. demonstrated that osteoporotic vertebral fragility fractures were strongly associated with worsening sagittal alignment [20]. Conversely, sagittal malalignment itself has also been reported to increase the likelihood of subsequent vertebral fractures [21,22]. In addition, initial vertebral fractures may predispose patients to both progressive spinal deformity and additional fractures [22,23]. Taken together, these findings suggest that although muscle mass and muscle strength may influence sagittal alignment, vertebral fractures likely play a more substantial role in sagittal imbalance among patients with osteoporosis. Therefore, prevention of vertebral fractures through appropriate osteoporosis treatment may contribute to preservation of spinal alignment.

The present study investigated the associations among muscle mass, BMD, and BMI in elderly patients with osteoporosis. Patients classified into groups with reduced muscle mass generally demonstrated lower BMD and BMI values, particularly among women. In addition, SMI showed positive correlations with BMD measurements in female participants. Previous reports have similarly described close relationships between SMM and bone density [6,7]. Furthermore, sarcopenia has been reported to occur frequently in patients with osteoporosis and osteoporotic fragility fractures [24,25]. Coin et al. also demonstrated that lower BMI was associated with reduced BMD in elderly individuals, especially in women [26]. In contrast, the relationship between muscle strength and bone-related parameters remains less clearly established. Based on the present findings, muscle mass may have a stronger association with BMD and BMI than muscle strength in elderly patients with osteoporosis.

In the present study, reductions in SMM and SMI were observed in the pre-sarcopenia and sarcopenia groups compared with the normal group, whereas patients with dynapenia generally maintained these parameters. In contrast, female patients in the dynapenia and sarcopenia groups demonstrated lower TMM values, suggesting a closer relationship between trunk muscle loss and impaired muscle strength. Although the association between TMM and muscle strength has not been fully clarified, previous studies have suggested that trunk musculature plays an important role in spinal alignment and clinical symptoms, including low back pain [16,27]. Hori et al. demonstrated that TMM was more strongly associated with sagittal alignment, ODI scores, and health-related quality of life than SMM [16]. Similarly, Yamamoto et al. reported that spinal kyphosis was more closely related to loss of TMM than to reductions in appendicular muscle mass [27]. These findings suggest that patients with dynapenia may retain overall SMM despite reductions in trunk muscle volume. Consequently, decreased TMM may contribute to impaired spinal balance and unfavorable clinical outcomes. Evaluation of osteoporosis should therefore include assessment of trunk musculature in addition to SMM.

A similar tendency was observed in analyses of low back pain-related outcomes. Patients in the dynapenia and sarcopenia groups demonstrated poorer JOABPEQ and ODI scores, whereas patients with pre-sarcopenia, who retained muscle strength, showed clinical outcomes comparable to those of the normal group. In male participants, grip strength was negatively associated with sagittal alignment parameters and also correlated with multiple low back pain-related outcome measures. Previous studies have identified low muscle mass, sagittal malalignment, elevated bone turnover, obesity, and aging as important contributors to low back pain in patients with osteoporosis [8]. Moreover, spinal sagittal malalignment has been reported in more than 70% of osteoporotic patients without vertebral fractures and is associated with worse low back pain-related outcomes [9]. These observations suggest that sagittal imbalance may substantially influence low back pain in osteoporosis.

Although vertebral fracture number did not differ significantly among groups in the present study, patients with reduced muscle strength consistently demonstrated poorer low back pain-related scores. Tanishima et al. similarly reported impaired low back pain-related outcomes in elderly patients with sarcopenia [28], while Sakai et al. demonstrated a high prevalence of sarcopenia among patients with chronic low back pain [29]. One possible mechanism underlying these associations involves chronic inflammation, as increased inflammatory cytokine activity has been linked to reductions in both muscle mass and muscle strength in older adults [30]. Collectively, these findings suggest that deterioration of muscle function may contribute directly to low back pain, highlighting the clinical importance of preserving muscle strength in patients with osteoporosis.

The present findings have potential clinical implications for the management of osteoporosis in older adults. While assessment of SMM is commonly performed, our results suggest that evaluation of muscle strength may provide additional information regarding functional status and low back pain-related disability. Incorporating grip strength assessment into routine clinical practice may help clinicians identify vulnerable patients who could benefit from targeted rehabilitation and exercise interventions.

Several limitations of this study should be acknowledged. First, all assessments were performed at the initial visit; however, many participants had already been receiving treatment for osteoporosis before enrollment. As a result, measurements such as BMD and bone turnover markers may have been influenced by ongoing pharmacological therapy. Second, vertebral fractures were evaluated only by number, whereas fracture severity and deformity were not analyzed in detail. Because vertebral deformity may substantially influence sagittal alignment and low back pain-related symptoms, this limitation should be considered when interpreting the present findings. Third, optimal adjustment methods for body composition measurements in patients with osteoporosis remain controversial. Many patients with osteoporosis have vertebral fractures and subsequent height loss, which may lead to underestimation of true body height. Consequently, height-adjusted indices such as SMI and TMI may be overestimated in some cases. To address this issue, both absolute muscle mass and height-adjusted muscle indices were evaluated in the present study. Nevertheless, differences in body constitution were not fully accounted for. Additional investigations are therefore required to establish more appropriate correction methods for body composition assessment in patients with osteoporosis. Fourth, because of the cross-sectional design of the present study, causal relationships between muscle mass, muscle strength, BMD, and low back pain-related outcomes could not be determined. Longitudinal studies are required to clarify whether deterioration of muscle mass or muscle strength directly contributes to the progression of osteoporosis-related disability and low back pain. Fifth, this study was conducted exclusively in Japanese patients with osteoporosis. Therefore, the generalizability of the present findings to populations with different ethnic, cultural, nutritional, and lifestyle backgrounds remains uncertain. However, Japan is recognized as one of the most advanced super-aging societies worldwide, and many countries are expected to experience similar demographic changes in the coming decades. Accordingly, although direct extrapolation should be interpreted with caution, the present findings may provide useful insights into future musculoskeletal health challenges associated with population aging. Future multinational studies are warranted to validate these findings across diverse populations. Finally, residual confounding may remain because several clinically important factors, including age, sex, BMI, primary versus secondary osteoporosis, and osteoporosis treatment status, were not fully adjusted for in the present analyses. Although baseline differences in age, sex, BMI, and osteoporosis subtype were summarized, future studies with larger sample sizes and detailed information on treatment history and comorbidities are needed to clarify the independent associations among muscle mass, muscle strength, BMD, and low back pain-related outcomes.

## Conclusions

Sarcopenia, pre-sarcopenia, and dynapenia were common among elderly patients with osteoporosis and were associated with distinct clinical characteristics. Reduced muscle mass was more closely related to BMD and body composition parameters, whereas reduced muscle strength was more strongly associated with low back pain-related disability and physical function. These findings suggest that muscle mass and muscle strength reflect different aspects of musculoskeletal health in elderly patients with osteoporosis. From a clinical perspective, assessment of muscle strength in addition to evaluation of muscle mass may provide additional information regarding functional status in elderly patients with osteoporosis. Grip strength assessment may help identify patients with distinct clinical characteristics despite preserved muscle mass. As Japan is one of the most advanced super-aging societies worldwide, these findings may provide useful insights into musculoskeletal health challenges that are expected to become increasingly important in aging populations globally. However, because of the cross-sectional design of the present study, causal relationships cannot be established, and further longitudinal and multinational studies are warranted.
